# Validity and reliability testing of the Toddler and Infant (TANDI) Health Related Quality of Life instrument for very young children

**DOI:** 10.1186/s41687-020-00251-4

**Published:** 2020-11-09

**Authors:** Janine Verstraete, Lebogang Ramma, Jennifer Jelsma

**Affiliations:** 1 Division of Medicine, Department of Paediatrics and Child Health, Klipfontein Road, Rondebosch, Cape Town, 7700 South Africa; 2 Division of Physiotherapy, Faculty of Health and Rehabilitation Sciences, Cape Town, South Africa; 3 Division of Communication Sciences and Disorders, Faculty of Health and Rehabilitation Sciences, Cape Town, South Africa

**Keywords:** Child, Infant, Toddler, Pre-schooler, Health, Health-related quality of life, HRQoL, Proxy, Outcome measure, EQ-5D-Y

## Abstract

**Background:**

Despite the high burden of disease in younger children there are few tools specifically designed to estimate Health Related Quality of Life (HRQoL) in children younger than 3 years of age. A previous paper described the process of identifying a pool of items which might be suitable for measuring HRQoL of children aged 0–3 years. The current paper describes how the items were pruned and the final draft of the measure, Toddler and Infant (TANDI) Health Related Quality of Life, was tested for validity and reliability.

**Methods:**

A sample of 187 caregivers of children 1–36 months of age were recruited which included children who were either acutely ill (AI), chronically ill (CI) or from the general school going population (GP). The TANDI, an experimental version of the EQ-5D-Y proxy, included six dimensions with three levels of report and general health measured on a Visual Analogue Scale (VAS) from 0 to 100. The content validity had been established during the development of the instrument. The TANDI, Ages and Stages Questionnaire (ASQ), Faces, Leg, Activity, Cry, Consolabilty (FLACC) or Neonatal Infant Pain Scale (NIPS) and a self-designed dietary information questionnaire were administered at baseline. The TANDI was administered 1 week later in GP children to establish test-retest reliability. The distribution of dimension scores, Cronbach’s alpha, rotated varimax factor analysis, Spearman’s Rho Correlation, the intraclass correlation coefficient, Pearson’s correlation, analysis of variance and regression analysis were used to explore the reliability, and validity of the TANDI.

**Results:**

Concurrent validity of the different dimensions was tested between the TANDI and other instruments. The Spearman’s Rho coefficients were significant and moderate to strong for dimensions of activity and participation and significant and weak for items of body functions. Known groups were compared and children with acute illness had the lowest ranked VAS (median 60, range 0–100), indicating worse HRQoL. The six dimensions of the TANDI were tested for internal consistency and reliability and the Cronbach’s α as 0.83. Test-retest results showed no variance for dimension scores of movement and play, and high agreement for pain (83%), relationships (87%), communication (83%) and eating (74%). The scores were highly correlated for the VAS (ICC = 0.76; *p* < 0.001).

**Conclusion:**

The TANDI was found to be valid and reliable for use with children aged 1–36 months in South Africa. It is recommended that the TANDI be included in future research to further investigate HRQoL and the impact of interventions in this vulnerable age group. It is further recommended that future testing be done to assess the feasibility, clinical utility, and cross-cultural validity of the measure and to include international input in further development.

## Background

According to the World Health Organization (WHO) there were approximately 6.2 million deaths globally in children and adolescents and of these deaths, 5.3 million occurred in children under 5 years of age, many of which could have been prevented [1]. In addition, under-fives contributed approximately 20% to the global burden of disease (in 2017) [2]. Despite the disease burden remaining disproportionately higher in the youngest members of society, there is only one preference based measure available for assessing the Health Related Quality of Life (HRQoL) of these most vulnerable younger children, the Health Status Classification System for Pre-School Children (HSCS-PS), however to date a utility score for the measure has not been developed [3–5]. Furthermore, the HRQoL measures which are currently available for this age group do not appear to have followed the guidelines of the Food and Drug Administration (FDA) [6, 7], the International Society for Pharmacoeconomics and Outcomes Research (ISPOR) [8] and other authorities [3, 5, 9–11]. These suggest that measures for young children be developed based on a sound conceptual model and that dimensions should report on observable behaviour. Observer-Reported Outcomes Measures (ObsRO) enables the observer to report on behaviour that he or she has seen, rather than having to infer what experienced HRQoL of the child is, based on their own subjective assessment [8].

As the childhood burden is often highest in resource constrained contexts, there is a clear need for a reliable, valid measure of HRQoL in younger children which is amenable for use in cost utility analysis. The Proxy version of the EQ-5D-Y appeared to perform reasonably well for physical dimensions, with some caveats, in children from three to 5 years of age [12]. However, there were issues of content validity identified, particularly in the younger age categories with recommendations for changes in dimensions for this age group. It was however evident that the Proxy EQ-5D-Y could not be used in children younger than 3 years. The need for a new measure for children under 3 years of age was further highlighted in the results of the systematic literature review and cognitive debriefing of caregivers of young children discussed elsewhere [3]. In response to this need, the authors set out to develop a new generic HRQoL measure, specifically for children under 3 years to be completed by proxy; the Toddler and Infant (TANDI) HRQoL measure. A systematic review of the literature identified the EuroQoL Youth version, the EQ-5D-Y, as the best model upon which to base the structure of the measure [3]. It is a simple measure which has been translated and validated in many countries, including Japan [13], Germany, Sweden and South Africa and across the globe [14]. The EQ-5D-Y is amenable to valuation and a standard protocol has now been developed to produce utility weights for children [15]. The formatting, layout, response options and time frame and the use of the Visual Analogue Scale (VAS) to assess global HRQoL were subsequently used in the presentation of the TANDI dimensions. The TANDI was then developed as an experimental version of an EQ Proxy with permission from the EuroQoL Research Foundation. The item bank was based on a systematic review of existing HRQoL measures for young children, cognitive interviews with caregivers of young children and a Delphi study with experts in the field of child health and HRQoL. The process of developing the item bank and age range for the measure has been previously described [3].

The original 11 dimensions were pruned, as described under the Methodology section, and the remaining six dimensions performed well on further testing were retained for the final measure. The EQ-5D-Y instructions for proxy completion were provided on the front page with a clear explanation that the dimensions should be completed from the viewpoint of the proxy not the child. The assessment of general health was scored on a Visual Analogue Scale (VAS) between 0 (worst imaginable health) and 100 (best imaginable health) and the layout and wording from the EQ-5D-Y proxy was retained.

The aim of this study was to test the validity and reliability of the TANDI in young children and infants across a range of ages and health conditions.

## Methods

A cross-sectional non-probability design was used to determine the validity of the TANDI. Longitudinal data was collected from a group of children from the general population for test retest reliability.

### Participants

The participants for this study included the caregivers of AI and CI children aged 1–36 months drawn from in-patients and out-patients respectively at a children’s hospital in Cape Town, South Africa. Participants also included caregivers of GP children attending several day care centres and toddler play groups in Port Elizabeth and Cape Town, South Africa. Caregivers had to be proficient in English as the prototype instrument was in English and would not be translated into other languages before validation. As English is one of the eleven official languages in South Africa [16] and the majority of education is in English [17], it was anticipated that most caregivers would have adequate command of the language to allow for participation. Caregivers of children who were admitted to the Intensive Care Unit, terminally ill or who were born prematurely and had not yet reached their corrected age of birth were excluded from the study. The sample size was calculated to allow factor analysis and it has been suggested as a rule of thumb, that at minimum ten observations is needed per variable [18]. The TANDI has six dimensions and a sample size of 60 would thus be needed in each of the health condition groups. The study aimed to compare three groups of children (AI, CI and GP) thus a minimum sample of 180 participants was needed.

### Procedure

Ethical approval from the University Human Research and Ethics Committee and permission from the children’s hospital and day-care centres was obtained. All caregivers of children attending the day-care centres and play groups were sent a detailed description of the study before data collection commenced. The research packs were sent home in the child’s school bag and caregivers who consented to participate were requested to return the envelope, sealed, with the completed research pack after 3 days. The research pack consisted of: information regarding the study and informed consent, demographic and medical information, TANDI, ASQ Parent Report Form, FLACC (for children aged 2–36 months) or NIPS pain scale (for children aged 1–2 months) and Dietary Information. The order of the questionnaires was standardised for all participants. All instruments were self-complete and caregiver training for completion of questionnaires was not required. The caregivers who participated were requested to complete a second TANDI measure and return it to school in the sealed envelope after 1 week. The time period was selected as the health of the GP children was not expected to change in this period and it ensured that caregivers did not remember what they answered on the first administration [19]. Only caregivers of children attending day care centres were asked to participate in the completion of a second copy of the TANDI as the play groups did not meet regularly.

Caregivers of AI children were recruited from the in-patient wards of a children’s hospital. The recruitment process was done systematically throughout the hospital according to ward and cubicle number. Caregivers of CI children were recruited from the waiting rooms of specialist clinics at the children’s hospital. Caregivers completed the research pack in a private room or in the case of AI children at the bedside if they preferred.

### Measures

#### TANDI

Testing of the Alpha Draft was done with 101 caregivers of children who were acutely ill or from the general population [3]. The results were subjected to psychometric testing and there were four dimensions that were not consistent with the other dimensions and removed. The Beta draft was then developed and tested with 60 caregivers of AI children for similar psychometric testing including: item acceptability, ceiling and floor effects, reliability and internal consistency, proportion of problems reported across age groups and effect of the dimension on the general health score measured on a Visual Analogue Scale (VAS).

**Fig. 1:**
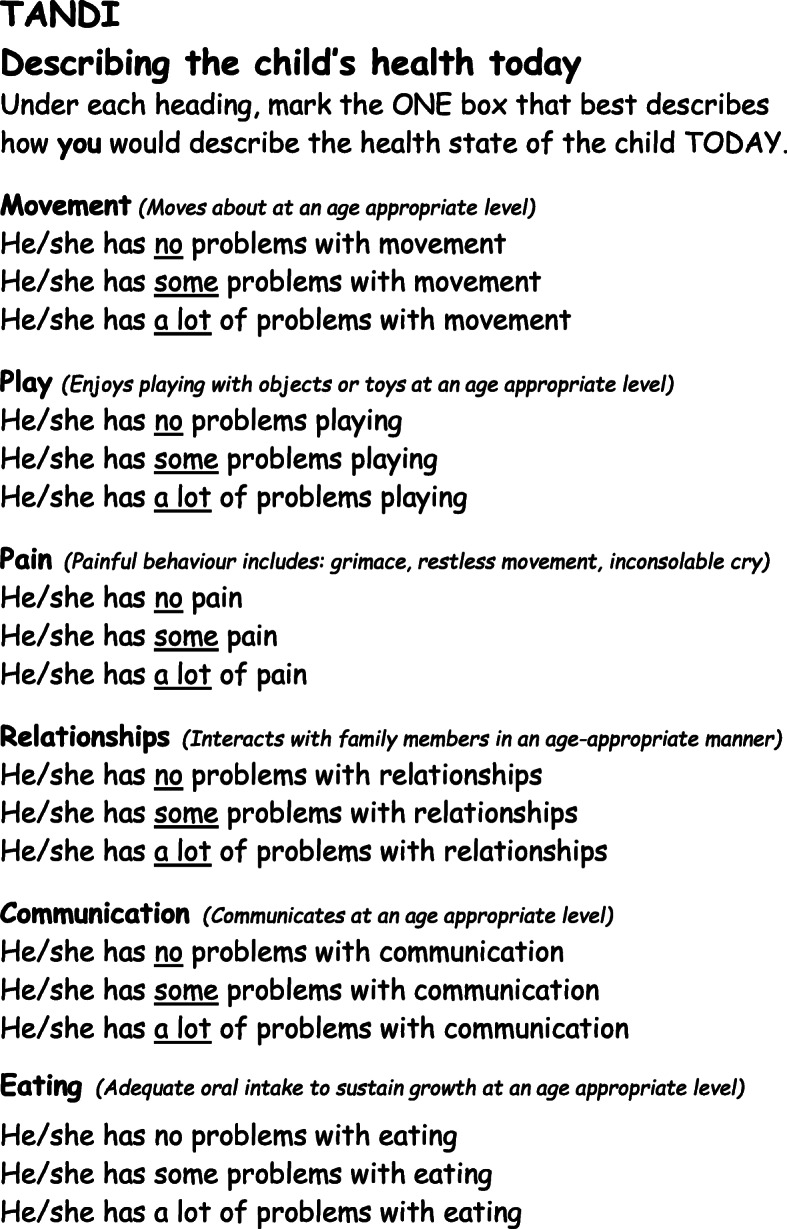
Example of TANDI items and wording

The Alpha draft of the TANDI consisted of 10 dimensions, scored on a three level likert scale, namely: behaviour, communication, eating, independence (helping with daily activities), play, mood (controlling emotions), movement, pain, relationships and sleep. The eleventh dimension of general health was scored on a VAS from 0 to 100 [3]. After the removal of the four dimensions and modifications in wording of the dimensions, the Beta draft thus consisted of six dimensions assessed on three level Likert scale as used in the EQ-5D-Y proxy version: no problems, some problems or a lot of problems. The layout, font, response options, VAS and recall period of the EQ-5D-Y-3 L Proxy were utilised but the dimensions included were developed from the ground up [3]. The instructions included in the EQ-5D-Y Proxy version were also included. The descriptors of the dimensions included in the Beta Draft and final version of the TANDI are included in Fig. 1. The measure has a maximum Flesch-Kincaid readability level of seven to ensure comprehensibility [20]. The Beta draft instrument was found to be multi-dimensional with two distinct factors with good internal consistency and reliability and was thus retained as the final measure for validity and reliability testing.

#### The Ages and Stages Questionnaire (third edition) (ASQ)

The ASQ was chosen to test concurrent validity. It is a caregiver-completed questionnaire to monitor development in children aged 1 month to 5 years of age [21, 22] and is valid and reliable internationally [21, 23–25]. There are 21 age specific questionnaires each comprising of 30 developmental items which are categorized into five different domains namely: communication, gross motor, fine motor, problem solving and personal social each scored on three levels: yes, sometimes and not yet [21]. Answers of yes, sometimes, and not yet are awarded ten, five and zero points respectively. The total score of 60 for each domain has two cut-off points which categorises domain scores as: the development of the child appears on par with developmental norms (score of 0–15); the development of the child is below the cut-off for normal development and needs monitoring (score of 20–30) and that the child needs to be assessed professionally (score of 35–60) [21, 22]. The ASQ has been found to be valid and reliable internationally [21, 23–25].

#### FLACC (face, leg, activity, cry, consolability) pain scale

For children who are not able to subjectively rate their pain, due to lack of cognitive and verbal skills, observation of pain behaviour is a validated approach of assessing pain [26]. The FLACC Scale is an observational behaviour tool which has been validated in children from 2 months to 7 years and is used widely in the clinical setting [26–31]. The scale considers typical pain behaviour in the face, legs, arms, activity, crying and consolability and scores each item from 0 to 2 [26–31]. A total summed score of zero indicates no pain, a score 1–3 indicates mild discomfort or pain, a score of 4–6 indicates moderate discomfort or pain and a score of 7–10 indicates severe discomfort or pain [27–30]. Mild and moderate discomfort and pain were combined for comparison to some pain reported on the TANDI.

#### Neonatal Infant Pain Scale (NIPS)

The Neonatal Infant Pain Scale (NIPS) is an observational measure for assessment of pain in children below 2 months. The items are similar to the FLACC scale and use of the measure does not require any additional training to complete [32–34]. The child’s behaviour of facial expression, cry, breathing pattern, activity in their arms and legs, and their state of arousal is scored between 0 and 1, except for the presence of cry which was scored from 0 to 2 [32]. A summed total score of zero indicated no pain, a score of 1–3 indicates mild pain and a score of 4–7 indicated severe pain.

#### Self-designed dietary information questionnaire

A literature search of dietary and eating assessment tools revealed three main instruments used in children: dietary record, 24 h dietary recall and food-frequency questionnaire [35–37]. These assessment methods had high respondent burden and the 24 h dietary recall required training of an interviewer [36, 37]. The assessment tools available were complex in nature and care needed to be taken to ensure that the measure used recorded issues relating to eating rather than the family’s food security. Thus, due to the lack of an assessment tool evaluating eating in general as opposed to nutritional status the decision was to include questions about the child’s eating and drinking for the time period ‘today’ for comparison to the dimension included on the TANDI. The questions were related to the amount of nutrition they were receiving orally in terms of: whether they were bringing up any of their food or milk, if they were eating or drinking as much as usual, the same amount as other children their age and at regular time intervals. Their ability to feed orally in relation to their competence with chewing/sucking and swallowing, gagging on food and tiring during eating was also assessed. The ability to eat orally was differentiated by asking whether an alternative feeding route via gastrostomy or intravenous infusion was used. The eight items were answered as yes/no and scored 1 point each with a higher score indicating a better eating ability. A score of 7–8 indicated a good eating ability, 4–6 indicated moderate eating ability and a score of 1–3 indicated poor eating ability. The questionnaire was content validated by an independent researcher, with good understanding of paediatric nutrition and feeding.

#### Demographic and medical information

The survey also included background questions to record age, gender, diagnosis, and relationship of caregiver to child.

### Data management and analysis

Age range of the children for inclusion was 1–36 months. To ensure that the instrument was applicable to children across this age band, three age groups were assessed during data analysis. The age groups were divided according to the child’s birthday and included: 1–12 months (1 month and 0 days to 11 months and 30 days); 12–24 months (12 months and 0 days to 23 months and 30 days) and 24–36 months (24 months and 0 days to 36 months and 0 days). The assessments of the measurement properties is described in detail in Table 1, the level of statistical significance was set at *p* ≤ 0.05. Statistical analysis was performed in IBM SPSS Statistics Version 26 and TIBCO Statistica version 13.

**Table 1:** Measurement Properties Tested

Domain	Measurement Property	Analysis
Distribution Characteristics	Floor and Ceiling effect	The distribution of frequency of dimension scores across condition groups was used to determine the distribution characteristics in terms of ceiling and floor effects (i.e large numbers of respondents reporting no problems or severe problems respectively, in each dimensions). The individual dimensions were considered, by the authors, to have a floor or ceiling effect if reporting exceeded 70%.
Measurement Structure	Factor Analysis	Rotated Factor Analysis was used to examine the structure of the questionnaire and determine the variance each of the factors contributed to the scale.
Reliability	Internal Consistency	Reliability and internal consistency of the dimensions was established through Cronbach’s Alpha. A Cronbach’s Alpha value of greater than 0.7 is considered acceptable [38].
	Test-retest reliability	Test-retest reliability was calculated, in a sub-set of GP children, for dimensions with percentage agreement and intraclass correlation coefficient (ICC) for the VAS score. An ICC of > 0.7 was considered reliable [39].
Validity	Known-group validity	The known group validity was established from the significance of chi-square results of dimension scores across AI, CI and GP children and one-way ANOVA and post hoc analysis of the VAS scores between AI, CI, and GP children. The dimensions were assessed for their equivalence across the age groups through the proportion of no problems which were reported and the 95% confidence intervals (CIs). It was hypothesised that the general health VAS score would distinguish between healthy toddlers and infants from the general population and those with acute and chronic health conditions based on previous findings from the EQ-5D-Y on older children [40, 41] and testing of the PedsQL Infant Scales [42]. It was anticipated that dimension scores would be similar to that reported for the EQ-5D-Y by Scott et al. (2017) and AI children would report the most problems across dimensions of movement, play, pain and eating; CI children would report more problems on the on dimensions of relationships and communication and some problems with movement; GP children would have ceiling effects for all dimensions with similar reporting of pain in GP and CI children [40].
	Concurrent Validity	The concurrent validity of the TANDI dimensions and the associated domain scores on the ASQ, FLACC scale or NIPS and Dietary Information was calculated by Sperman’s Rho. Correlation values were interpreted according to Dancey and Reidy guidelines with correlations from 0.1 to 0.3 were considered weak, 0.4 to 0.6 moderate and correlations of 0.7 or above as strong [43]. The performance of the dimensions across age groups was assessed to ensure that the measure was valid across the age range and different versions would not be needed for each age group.
	Performance of the TANDI General Health Question measured on the VAS	Regression Analysis of the VAS score was used to determine the effect that the dimension scores had on the general health. Outliers with residual scores > 2 Standard Deviations from the mean were excluded for regression analysis.

## Results of validity and reliability testing

### Descriptive statistics

Research packs were sent to 112 caregivers of GP children inviting them to participate in the study. Caregivers of 67 children consented and returned the research packs. A second copy of the TANDI was sent to 46 GP children attending day-care centres of which 23 returned completed measures. All the caregivers of AI and CI children who were approached and met the inclusion and exclusion criteria consented to participate and completed the study.

Age Bracket was significantly associated with health condition group (Chi-sq 51.88; *p* < 0.001). There were a higher number of children in the 1–12-month group for AI and in the 24–36 month group for GP. Gender was not significantly associated with condition group, although there were double the number of males to females in the CI group (Chi-sq-4.34; *p* = 0.114). Most caregivers across condition groups were mothers and relationship of caregiver to child was not significant across condition groups (Chi-sq = 15.54 and *p* = 0.114) (Table 2).

**Table 2:** Descriptive statistics of the sample

Age group	AI (***n*** = 60)	CI (***n*** = 60)	GP (***n*** = 67)	Total (***n*** = 187)
	N (%^**a**^)	N (%^**a**^)	N (%^**a**^)	N (%^**b**^)
**Relationship of caregiver to child**				
Mother	55 (92)	49 (82)	65 (97)	169 (90)
Father	3 (5)	5 (8)	1 (1.5)	9 (5)
Other	2 (3)	6 (10)	1 (1.5)	9 (5)
**Gender of Child**				
Female	31 (52)	20 (33)	31 (46)	82 (44)
**Age Group of the Child**				
1–12 months	38 (63)	14 (23)	6 (9)	58 (31)
12–24 months	12 (20)	23 (38.5)	20 (30)	55 (29)
24–36 months	10 (17)	23 (38.5)	41 (61)	74 (40)
**Primary Diagnosis of the Child**				
None	0	0	59 (88)	59 (16)
Neurodevelopmental	3 (5)	33 (55)	2 (3)	38 (20)
Surgical	15 (25)	1 (2)	0	16 (9)
Pneumonia	11 (18)	0	1 (1)	12 (6)
Haematology/Oncology	0	10 (17)	0	10 (5)
Congenital Heart Disease	8 (13)	1 (2)	0	9 (5)
Upper Airway Obstruction with Tracheostomy	6 (10)	3 (5)	0	9 (5)
GIT	3 (5)	2 (3)	0	9 (5)
Diarrheal disease	6 (10)	0	1 (1)	7 (4)
Allergy/Asthma	1 (2)	3 (5)	2 (3)	6 (3)
Liver Disease	4 (7)	2 (3)	0	6 (3)
Other	3 (5)	5 (8)	2 (3)	10 (5)

*N* = 187 ^a^% of condition group, ^b^% of total sample

The participants had a wide range of conditions with most of the GP children reported as not having any medical condition and many of the AI children were hospitalised for a range of surgical procedures including general surgery and neurosurgery (Table 2). The aetiologies for the CI children were complex and children often presented with multiple associated conditions/illnesses. Only the primary diagnosis of CI children has been reported. Neurodevelopmental concerns were the most frequently reported condition which includes cerebral palsy, developmental delay, and epilepsy. The category of other included eczema, malnutrition, breath holding spells, chronic lung disease and a sore throat.

There was no missing data for either the dimensions or VAS of the TANDI. All measures were completed in full except the FLACC pain scale which was not completed by seven caregivers of GP children.

### Factor structure, reliability and internal consistency of the TANDI

The overall reliability of the scale was good with α = 0.83 [38]. Dimensions of pain and eating were shown to increase reliability if removed. The item-rest correlation for pain (0.31) and eating (0.42) are also lower. This is in keeping with the factor analysis where pain and eating formed a second factor, body functions, on the scale accounting for 17% of the variance. The other dimensions all loaded on the first factor, activities and participation, and contributed to 55% of the variance.

#### Test-retest reliability

Test-retest reliability was only done in 23 GP children. There is was little variance in the dimension scores of the GP children participating in the test-retest of the TANDI. As the numbers were so small no statistical tests were done. The percentage of absolute agreement was movement =100%, play = 100%, pain = 83%, relationships = 87%, communication = 83% and eating =74%. The VAS re-test scores showed significant reliability (ICC = 0.76; *p* < 0.001).

### Performance of TANDI across condition groups

The scores of AI children were similar across all dimensions with the reporting of no problems ranging between 58% -73%. Caregivers of CI children tended to report extremes (no problems or a lot of problems) for all dimensions except pain. GP children reported ≥88% of no problems and ≤ 1% for a lot of problems for all dimensions except eating (no problems = 75%; a lot of problems = 3%) (Table 3). For every dimension the GP caregivers reported the greatest percentage of children with no problems and the smallest percentage with severe problems.

**Table 3:** Dimension Scores of the TANDI Across Condition Groups

Dimensions	AI (*n* = 60)	CI (*n* = 60)	GP (*n* = 67)	Total (*n* = 187)	AI v CI	AI v GP	CI v GP
	N (%)	N (%)	N (%)	N (%)	Chi-Sq	Chi-Sq	Chi-Sq
Movement							
1^§^	36 (60)	31 (52)	64 (96)	131 (70)	2.041	22.938**	30.450**
2^§^	8 (13)	14 (23)	3 (4)	25 (13)			
3^§^	16 (27)	15 (25)	0 (0)	31 (17)			
Play							
1^§^	35 (58)	36 (60)	64 (96)	135 (72)	0.524	24.324**	22.284**
2^§^	9 (15)	11 (18)	3 (4)	23			
3^§^	16 (27)	13 (22)	0 (0)	29 (16)			
Pain							
1^§^	44 (73)	53 (88)	60 (90)	157 (84)	12.743**	6.701*	0.474
2^§^	16 (27)	5 (9)	6 (9)	27 (14)			
3^§^	0 (0)	2 (3)	1 (1)	3 (2)			
Relationships							
1^§^	42 (70)	41 (68)	59 (88)	142 (76)	0.071	9.763*	10.202*
2^§^	8 (13)	9 (15)	8 (12)	25 (13)			
3^§^	10 (17)	10 (17)	0 (0)	20 (11)			
Communication							
1^§^	38 (63)	33 (55)	59 (88)	130 (70)	4.080	19.326**	22.304**
2^§^	13 (22)	9 (15)	8 (12)	30 (16)			
3^§^	9 (15)	18 (30)	0 (0)	27 (14)			
Eating							
1^§^	36 (60)	42 (70)	50 (75)	128 (68)	3.947	20.718**	7.797*
2^§^	5 (8)	8 (13)	15 (22)	28 (15)			
3^§^	19 (32)	10 (17)	2 (3)	31 (17)			
	Median (IQR)	Median (IQR)	Median (IQR)	ANOVA			
General Health (VAS)	60 (0 to 100)	77 (15 to 100)	90 (52 to 100)	F (2.18) = 15.65**	***p*** **= 0.699**	***p*** **< 0.001**	***p*** **< 0.001**

Bolded scores indicate significance, VAS is scored from 0 to 100 with a higher score indicating a better general health

1^§^ No problem, 2^§^ some problems, 3^§^ A lot of problems. * = *P* < 0.05, ** = *P* < 0.001

Ceiling effects were noted for AI children in the dimensions of pain (73%) and relationships (70%) and CI children for pain (88%) and eating (70%). All dimensions had ceiling effects for GP children.

There was a significant difference between AI and GP children for all dimensions. Similarly, there was a significant difference between CI and GP children for all dimensions except for pain. The only dimension that was significantly different between AI and CI children was pain.

Post Hoc Tukey Analysis of the General Health VAS scores similarly revealed that GP was significantly different to AI and CI (*p* < 0.01) but AI and CI were not different.

Sub-Analysis showed that AI children who reported problems with pain, reported problems across all other dimensions. CI children who reported problems with pain, reported problems with movement, communication, and play. GP children who reported problems with pain also reported problems with eating.

### Performance of the TANDI across age groups

Although the proportion of problems reported for dimensions of movement, play and communication are all increased for the 12–24-month age group, the 95% CIs overlap for all age-groups across all dimensions. There was no significant difference between VAS scores across age groups (F (2.18)=1847 (*p* = 0.161) (Table 4).

**Table 4:** Proportion of Problems Reported for TANDI dimensions for all children per age group

Age Groups	TANDI Dimensions						General Health	
	Mvt	Play	Pain	Rel	Comm	Eat	VAS	
1–12 months (*n* = 58)								
Proportion of Probs	0.31	0.28	0.17	0.21	0.24	0.38	Median	90
CI	0.21	0.18	0.10	0.12	0.15	0.27	IQR	65 to 95
	0.44	0.40	0.29	0.33	0.37	0.51		
12–24 months (*n* = 55)								
Proportion of Probs	0.38	0.33	0.18	0.22	0.38	0.24	Median	80
CI	0.27	0.22	0.10	0.13	0.27	0.14	IQR	70 to 90
	0.51	0.46	0.30	0.34	0.51	0.36		
24–36 months (*n* = 74)								
Proportion of Probs	0.23	0.24	0.14	0.28	0.30	0.32	Median	80
CI	0.15	0.16	0.08	0.19	0.21	0.23	IQR	50 to 95
	0.34	0.35	0.23	0.40	0.41	0.44		

VAS is scored from 0 to 100 with a higher score indicating a better general health. ANOVA for General Health VAS scores by age group: F (2.18)=1847 (*p* = 0.161)

*mvt* movement, *rel* relationships, *comm* communication

### Concurrent validity of the TANDI dimensions

There was strong agreement between TANDI dimensions of movement, play and eating with the comparable dimensions of the ASQ Gross motor, problem solving and dietary information. Pain showed moderate agreement with the FLACC and NIPS pain scale, there was significant, although weak, agreement with pain and dietary info supported by the fact that these two dimensions load on the same factor of body functions. There was moderate agreement between the dimensions loaded on the other factor representing activities and participation which include functions of activity and participation namely: movement, play, relationships, and communication (Table 5). There was however a divergent relationship between pain and dimensions representing activities and participation. A weaker relationship between eating and dimensions of activities and participation were noted.

**Table 5:** Spearman’s Rho Correlations of TANDI Dimensions

TANDI Dimensions	ASQ Gross Motor (*N* = 187)	ASQ Fine motor (*N* = 187)	ASQ Prob Solving (*N* = 187)	FLACC & NIPS (*N* = 180)	ASQ Personal & Social (*N* = 187)	ASQ Comm (*N* = 187)	Dietary Info (*N* = 187)
Movement	0.78**	0.67**	0.67**	−0.06	0.66**	0.64**	0.34**
Play	0.69**	0.69**	0.69**	−0.05	0.71**	0.63**	0.34**
Pain	0.12	0.13	0.09	0.47**	0.14	0.10	0.25**
Relationships	0.45**	0.51**	0.54**	0.04	0.55**	0.51**	0.31**
Communication	0.55**	0.64**	0.65**	0.03	0.70**	0.74**	0.26**
Eating	0.32**	0.27**	0.21**	0.11	0.36**	0.25**	0.76**

Seven caregivers did not complete the FLACC/NIPS pain scales

* = *P* < 0.05, ** = *P* < 0.001. Bold indicates strong agreement

### Performance of the TANDI general health question measured on the VAS

Multiple regression analysis with the VAS as dependent variable and dummy variables representing the different levels of the dimensions accounted for 35% of the variance. The model improved and accounted for 45% of the variance once nine outliers, with residual scores > 2 Standard Deviations from the mean, were excluded.

Coefficients of Movement 2^§^, Pain 2^§^, Relationships 3^§^, Eating 2^§^ and 3^§^ all significantly detracted from the VAS score. All the other dimensions detracted from the VAS score and a lot of problems detracted more than some problems for all dimensions (Table 6).

**Table 6:** Regression Analysis of the TANDI VAS Score and Dimension Scores

	b*	Std. Err. Of b*	b	Std. Err. Of b	t(164)	*p*-value
Intercept			88.74	1.46	60.69	0.000
Movement 2^§^	−0.17	0.07	−9.36	3.73	−2.51	***0.013***
Movement 3^§^	−0.18	0.09	−10.30	5.22	−1.97	0.050
Play 2^§^	−0.03	0.07	−1.64	4.02	−0.41	0.685
Play 3^§^	−0.07	0.11	−4.26	5.99	−0.71	0.477
Pain 2^§^	−0.13	0.06	−6.92	3.39	−2.04	***0.043***
Pain 3^§^	−0.07	0.06	−10.90	8.75	−1.25	0.215
Relationships 2^§^	−0.03	0.07	−1.92	3.79	−0.51	0.614
Relationships 3^§^	−0.16	0.08	−11.64	5.74	−2.03	***0.044***
Communication 2^§^	−0.04	0.07	−2.21	3.67	−0.60	0.548
Communication 3^§^	−0.07	0.09	−4.42	5.11	−0.87	0.388
Eating 2^§^	−0.12	0.06	−6.38	3.12	−2.05	***0.042***
Eating 3^§^	−0.29	0.07	−15.11	3.51	−4.30	***< 0.001***

*N* = 178 1^§^ No problem, 2^§^ Some problems, 3^§^ A lot of Problems

Adjusted *R*^*2*^ = 0.45(*n* = 178). Bolded Scores indicate significance

b* denotes standardized Beta regression coefficients; b denotes non-standardized Beta regression coefficients

## Discussion

The results of this research suggest that the newly developed TANDI is feasible, demonstrates internal consistency reliability, test-retest reliability and validity. It also appears that the TANDI is a multi-dimensional instrument that allows for generic HRQoL to be assessed between 1 and 36 months using the same six dimensions namely, movement, play, pain, relationships, communication and eating. Performance of the general health VAS score performed as hypothesised and similarly to the PedsQL Infant Scale Total Score [42] in that it distinguished between toddlers and infants from the general population and those with acute and chronic health conditions. There were further no age-related differences in the scoring of general health. Thus, as an experimental version of the EQ-5D-Y proxy version, the TANDI could articulate well with the existing EQ-5D-Y versions, possibly allowing changes in HRQoL to be tracked across the lifespan on similar dimensions. Furthermore, by retaining the structure of the EQ-5D-Y, specifically the recall period of Today, three levels of report and limited number of dimensions the authors have ensured that the measure would be amenable to elicitation of preference weights in the future.

As the ICF was chosen as the guiding conceptual framework, it was necessary to ensure that the identified dimensions were representative of both body functions and the activities and participation categories [44]. This multi-dimensionality was confirmed through the results of the rotated factor analysis with the dimensions of movement, play, pain and relationships representing activities and participation and eating and pain representing body functions. In line with current recommendations for instrument development [6–8, 45] face and content validity were ensured as caregivers and experts in the field were included in the developmental process as described previously [3]. The FDA and ISPOR both recommend that measures are based on observable behaviour [6–8] and to ensure observability descriptions of the dimensions were tested in the Alpha and Beta Drafts of the measure. The descriptions included in the Alpha draft were criterion referenced with examples of developmentally appropriate tasks for each age group except for pain where painful behaviours were described. Testing of the Alpha draft however concluded that the criterion-based dimensions descriptors were not useful to caregivers. Apart from the pain dimension, caregivers used norm referencing and compared the behaviour of other children of a similar age in selecting responses. As such the Beta Draft retained the descriptors for pain whereas the other descriptions were norm referenced as indicated in Fig. 1 (“moves about at an age appropriate level”). This was well accepted on testing of the Beta Draft and was thus retained for the final measure. Norm referencing of each dimension helped to ensure that the proxy respondent compared the observable behaviour of the child to that of observable characteristics of other children of the same age. We acknowledged that pain behaviour is not easily observable and typical behaviour associated with pain was included based on literature (painful behaviour includes: grimace, restless movement, inconsolable cry) [27, 28, 33, 46, 47].

The internal consistency reliability of the six dimensions included on the TANDI was good and surpassed the recommended minimum alpha co-efficient of 0.70 [38]. There were no missing responses on the TANDI suggesting that caregivers could easily provide data regarding their child’s HRQoL. The TANDI dimensions performed well with no floor effects for children with acute and chronic conditions. As the instrument only has three levels of report, floor effects in children with a health condition would be problematic and would have indicated that the levels were not adequately responsive to health condition.

Known group validity was determined by comparing dimension scores between AI, CI and GP children and the results indicate that dimension scores and the general health VAS score may be used to examine HRQoL in infants and toddlers related to health condition. Dimension scores performed as hypothesised with greater frequencies of problems with play, pain and eating reported in AI children and marginally more problems reported for dimensions of relationship and communication for CI children. There were however no significant differences between these dimension scores for the AI and CI groups although there were more problems with movement in the CI group than the AI group. This could be attributed to the large group of children with neurodevelopmental concerns in the CI group which results in delays across all dimensions except for pain, which remained significantly higher in the AI group. The dimensions behaved as expected in the GP group with significantly less reporting of problems across all dimensions compared to the AI and CI group. The exception was the dimension of pain in which the frequency of reporting some or a lot of pain was similar in the CI and GP children. This may speak to the nature of chronic conditions where conditions of neurodevelopmental nature would not be associated with pain in this age group. As anticipated the GP group had ceiling effects for all dimensions and the frequency of reported problems in the other dimension scores are consistent with published results of the EQ-5D-Y in healthy groups of children [14, 41, 48–52]. The ceiling effects for pain and relationships in AI children and pain and eating in CI children may be due to the heterogeneity of condition groups in the respective samples.

Pain may have been under-reported as this dimension is inferred from behaviour rather than observed directly and the frequency of children reported to have no problems in this dimension was relatively high. However, the results were intuitively correct as the frequency of “no problems” did decrease in this dimension across the groups as might be expected, with GP, CI and AI reporting 90%, 88% and 73% respectively. This result is consistent with that of Scott et al. (2017) who found that older children attending main stream schools reported similar proportions of pain to those with chronic health conditions attending special schools, which included children with conditions of motor disability [40]. Sub-analysis showed that AI children who were reported as having pain also reported problems on other items. This may be attributed to painful procedures and the limitations of participation and activities associated with acute illness and surgery. Most of the AI children who were reported to have some problems with pain also reported problems with eating. The relationship between pain and eating is further seen in that they load on the same factor and that they show weak but significant agreement for concurrent validity. Problems with feeding or eating are common in children with 20–50% of typically developing children, and 70–89% of children with developmental disabilities reported as having feeding problems [53]. Gastroesophageal reflux is a common eating problem which causes pain or discomfort [53, 54] and could be contributing to the report of pain and eating problems most noted in the GP group. Reflux is present in more than 25% of children under 18 months [54] this could be reflected in the larger confidence interval seen in the proportion of problems reported in the youngest age group. More severe problems with feeding was associated with illness as AI had the highest report of a lot of problems with eating. The more severe problems with eating in the AI group could be associated with pathological causes of difficulty with eating associated with difficulties with swallowing, severe gastroesophageal reflux disease, vomiting, diarrhoea and failure to thrive [54].

The results establish concurrent validity of the TANDI as dimension scores showed strong relationships with standardized measures in comparable domains. The result was similar to the validity testing of the HSCS-PS where concurrent validity was higher for motor-related areas of function and lower for less tangible functions such as vision and pain/discomfort [55]. There was strong agreement between dimensions with the same construct on the ASQ and moderate agreement on movement, play, relationships and communication with the other ASQ developmental domains highlighting the interdependence of motor, cognitive, emotional and social development in this young age group [56, 57]. These dimensions loaded on the same factor and contributed to the internal consistency of the measure further strengthening that they are interrelated. This supports the conceptual framework of the ICF where children develop activities such as movement and communication to become independent and develop relationships and participate in activities such as play [56]. The dimensions of eating and pain, classified as a body function on the ICF, did not show good agreement with dimensions of activities and participation. They are not dependent on each other for development and thus showed poor agreement with each other. The dimension of pain on the TANDI had a weak, significant agreement with dimensions on the FLACC or NIPS pain scale. Although observable descriptors were included on the TANDI for the dimension of pain it highlights the complex nature of pain and the difficulty in assessment, especially in the non-verbal child [58]. In the absence of a concise, parent report feeding questionnaire a self-design questionnaire was developed. The results of the questionnaire demonstrated significant, high agreement with the dimension of eating on the TANDI and had poor agreement other dimensions unrelated to eating. These results indicate that the questionnaire was sensitive and specific in reporting of eating problems in these children.

With regard to content validity, the dimensions included on the TANDI appear to account for a large proportion (45%) of the variance in the perceived general health score, the VAS. The results were logically consistent, and problems reported on each of the dimensions detracted from the general health scores and a lot of problems detracted more than some problems. A lot of problems with eating had the biggest impact on general health of the child, followed by a lot of problems with relationships, pain and movement. Eating/feeding is a frequent, important occurrence in the life of an infant and toddler and a large focus of attention for parents [59] with concerns over feeding being one of the most frequent concerns on parental online message boards [60]. The eating experience is critical for providing sustenance needed for good health and provides an opportunity for social interaction and development of relationships [59], which the results of this study indicate is the second greatest detractor from general health. Parental concerns over eating/feeding in infants and toddlers include adequate nutrition, weaning from the breast, independent eating [60], picky eating behaviours and refusal to try new foods [61].

Although the test-retest result of the TANDI showed good agreement the inclusion of only GP children limited the variance of response and the subsequent results obtained. It is recommended that future studies are needed to further establish the test-retest reliability include CI children.

Due to the rapid development that takes place in the infant and toddler phase, measures functioning such as the Bayley Scales of Infant Development [62] and Ages and Stages Questionnaire [63] have a number of different items or questionnaires depending on the age of the child. Thus, it was important to establish whether the dimensions included on the TANDI could measure HRQoL across the ages from 1 to 36 months. Age group analysis for each dimension was performed to ensure that there were no age-related differences in the proportion of reporting of problems between age groups. We suggest that the TANDI can be considered a ‘one-size-fits-all’ instrument as the dimensions and VAS performed well across all ages. It is noted that although the confidence intervals overlapped across age groups, there was a higher proportion of problems for dimensions of movement, play and communication in the 12–24-month age group. This spike may reflect the health condition of the sample, rather than a symptom of differential age responses as conditions of cerebral palsy, developmental delay and epilepsy were most frequently reported in this age group. Notable developmental milestones of walking and talking develop between 12 and 24 months and delays in these milestones are noted with neurodevelopmental disability. In fact walking, floor mobility, stiffness and communication are the most frequently reported parental concerns in children with cerebral palsy under 2 years [64].

### Limitations of the study

The sample of convenience and not having information on non-participants from the GP group may limit the generalizability of the findings to other infants and toddlers. The heterogenous group of AI and CI children could limit the generalizability of known groups validity. It is recommended that future studies include known disease groups and or disease severity analysis to further establish construct validity.

The inclusion of GP children for reliability analysis on the test-retest of the instrument limited the analysis of the data and the subsequent results obtained. It is recommended that future studies wanting to evaluate the test-retest reliability include CI children with a shorter period between reports. This will increase the variance in the results obtained making analysis easier. External variables such as global impression measures could also be included in test-retest analysis to identify the subsample with minimal change in health across the test-retest period.

Cognitive debriefing after completion the final TANDI measures could have been useful to further establish the comprehensibility and acceptability of the measure. The study results are limited to English speaking caregivers of children who were AI, CI or GP and the results are thus not generalizable. It is recommended that future studies include caregivers from different cultural groups.

## Conclusion

This research indicates that the TANDI is a promising new addition to the instruments used to measure HRQoL in very young children. The final version of the TANDI was found to be valid and reliable for use with children aged 1–36 months in South Africa and we suggest that it be included in the assessment of children with health conditions within this context. However, further research is needed to establish the feasibility, clinical utility and cross-cultural validity of the measure in other languages and cultural contexts.

## Data Availability

The datasets used and/or analysed during the current study are available from the corresponding author on reasonable request. The TANDI is an experimental version of the EQ-5D-Y Proxy, permission for use needs to be made with the EuroQoL Research Foundation.

## Abbreviations

AI: Acutely Ill
ASQ: Ages and Stages Questionnaire
CIs: Confidence Intervals
CI: Chronically Ill
Comm: Communication
Eat: Eating
FDA: Food and Drug Administration
FLACC: Faces, Leg, Activity, Cry, Consolabilty
GP: General Population
HRQoL: Health Related Quality of Life
HSCS-P: The Health Status Classification for Pre-School Children
ICC: Intraclass correlation coefficient
ICF: International Classification of Functioning, Disability & Health
ISPOR: International Society for Pharmacoeconomics & Outcomes Research
Mvt: Movement
NIPS: Neonatal Infant Pain Scale
ObsRO: Observer-Reported Outcomes Measures
Rel: Relationship
TANDI: Toddler and Infant
VAS: Visual Analogue Scale
WHO: World Health Organization

## References

[1] World Health Organization (2019). Children: reducing mortality. Newsroom, Factsheets.

[2] Roser M, Ritchie H (2016). Burden of Disease.

[3] Verstraete J, Ramma L, Jelsma J (2020). Item generation for a proxy health related quality of life measure in very young children. Health and Quality of Life Outcomes.

[4] Janssens A, Rogers M, Thompson Coon J, Allen K, Green C, Jenkinson C (2015). A systematic review of generic multidimensional patient-reported outcome measures for children, part II: Evaluation of psychometric performance of english-language versions in a general population. Value in Health.

[5] Janssens A, Thompson Coon J, Rogers M, Allen K, Green C, Jenkinson C (2015). A systematic review of generic multidimensional patient-reported outcome measures for children, part I: descriptive characteristics. Value in Health.

[6] U.S Department of Health and Human Services Food and Drug Administration, Center for Drug Evaluation and Research, Center for Biologics Evaluation and Research, Health C for D and R (2009). Guidance for industry. patient-reported outcome measures: use in medical product development to support labeling claims guidance for industry. *Clinical/Medical Federal Register*, 1–39 Available from: https://www.fda.gov/regulatory-information/search-fda-guidance-documents/patient-reported-outcome-measures-use-medical-product-development-support-labeling-claims.

[7] U.S Food and Drug Administration (2018). Discussion Document for Patient-Focused Drug Development Public Workshop on Guidance 3: Select, Develop or modify fit-for-purpose clinical outcome assessments.

[8] Matza LS, Patrick DL, Riley A, Alexander J, Rajmil L, Pleil A (2013). Pediatric patient-reported outcome instruments for research to support medical product labeling: Report of the ISPOR PRO good research practices for the assessment of children and adolescents task force. Value in Health.

[9] Grange A, Bekker H, Noyes J, Langley P (2007). Adequacy of health-related quality of life measures in children under 5 years old: systematic review. Journal of Advanced Nursing.

[10] Eiser C, Morse R (2001). Can parents rate their child’s health-related quality of life? Results of a systematic review. Quality of Life Research.

[11] Mokkink LB, Terwee CB, Patrick DL, Alonso J, Stratford PW, Knol DL (2010). The COSMIN checklist for assessing the methodological quality of studies on measurement properties of health status measurement instruments : An international Delphi study. Quality of Life Research.

[12] Verstraete J, Jelsma J (2017). Performance of the EQ-5D-Y proxy in very young children.

[13] Shiroiwa T, Fukuda T, Shimozuma K (2019). Psychometric properties of the Japanese version of the EQ-5D-Y by self-report and proxy-report: reliability and construct validity. Quality of Life Research.

[14] Ravens-Sieberer U, Wille N, Badia X, Bonsel G, Burström K, Cavrini G (2010). Feasibility, reliability, and validity of the EQ-5D-Y: results from a multinational study. Quality of Life Research.

[15] Ramos-Goñi, J. M., Oppe, M., Stolk, E., Shah, K., Kreimeier, S., Rivero-Arias, O., et al. (2020;(0123456789). Available from). International valuation protocol for the EQ-5D-Y-3L. *Pharmacoeconomics*. 10.1007/s40273-020-00909-3. 10.1007/s40273-020-00909-3 32297224

[16] Lehohla P (2012). Census 2011 - Census in brief. Vols. 03–01–41, Statistics South Africa.

[17] South African Department of Basic Education. Department of Basic Education (2010). *The status of the Language of Learning and Teaching (LOLT) in South African Public Schools*, (p. 44). Pretoria: Department of Basic Education.

[18] Maccallum RC, Widaman KF, Preacher KJ, Hong S (2001). Sample size in factor analysis: the role of model error. Multivariate Behavioral Research.

[19] Macran S, Brooks R, Rabin R, de Charro F (2003). Test-retest performance of EQ-5D. The measurement and valuation of health Staus using EQ-5D: A European perspective.

[20] Marnell G. (2008). Measuring Readability Part 1: The spirit is willing but Flesch is weak. Southern Communicator, (14), 1–16.

[21] Squires J, Twombly E, Bricker D, Potter L (2009). ASQ-3 User’s Guide.

[22] Squires J, Bricker D, Potter L (1997). Revision of a parent-completed development screening tool: Ages and stages questionniare. Journal of Pediatric Psychology.

[23] Gollenberg AL, Lynch CD, Jackson LW, McGuinness BM, Msall ME (2010). Concurrent validity of the parent-completed Ages and Stages Questionnaires, 2nd Ed. with the Bayley Scales of Infant Development II in a low-risk sample. Child: Care, Health and Development.

[24] Filgueiras A, Pires P, Maissonette S, Landeira-Fernandez J (2013). Psychometric properties of the Brazilian-adapted version of the Ages and Stages Questionnaire in public child daycare centers. Early Human Development.

[25] Kerstjens JM, Bos AF, ten Vergert EMJ, de Meer G, Butcher PR, Reijneveld SA (2009). Support for the global feasibility of the ages and stages questionnaire as developmental screener. Early Human Development.

[26] Herr K, Coyne PJ, Manworren R, McCaffery M, Merkel S, Pelosi-Kelly J (2006). Pain assessment in the nonverbal patient : position statement with clinical practice recommendations. American Society for Pain Management Nursing.

[27] Manworren R, Hynan L (2003). Clinical validation of FLACC: Preverbal patient pain scale. Pediatric Nursing.

[28] Merkel S, Voepel-Lewis T, Malviya S (2002). Pain Assessment in infants and young children: The FLACC scale. AJN The American Journal of Nursing.

[29] Malviya S, Voepel-Lewis T, Burke C, Merkel S, Tait AR (2006). The revised FLACC observational pain tool: improved reliability and validity for pain assessment in children with cognitive impairment. Paediatric Anaesthesia.

[30] Merkel SI, Voepel-Lewis T, Shayevitz JR, Malviya S (1997). The FLACC: a behavioral scale for scoring postoperative pain in young children. Pediatric Nursing.

[31] Voepel-Lewis T, Zanotti J, Dammeyer JA, Merkel S (2010). Reliability and validity of the face, legs, activity, cry, consolability behavioral tool in assessing acute pain in critically ill patients. American Journal of Critical Care.

[32] Gallo A-M (2003). The fifth vital sign: Implementation of the neonatal Infant pain scale. Journal of Obstetric, Gynecologic, and Neonatal Nursing.

[33] Hudson-Barr D, Capper-Michel B, Lambert S, Palermo TM, Morbeto K, Lombardo S (2002). Validation of the pain assessment in neonates (PAIN) scale with the Neonatal Infant Pain Scale (NIPS). Neonatal Network.

[34] Duhn LJ, Medves JM (2004). A systematic integrative review of infant pain assessment tools. Advances in Neonatal Care.

[35] Colditz GA, Rockett HR (1997). Assessing diets of children and adolescents. The American Journal of Clinical Nutrition.

[36] Frances E, Thompson AFS (2008). Dietary assessment methodology. Nutrition in the Prevention and Treatment of Disease.

[37] Thompson, F. E., & Byers, T. (1994). Dietary Assessment Methodology. *Am Inst Nutr J Nutr, 124*, 2245S–2317S. 10.1093/jn/124.suppl_11.2245s 7965210

[38] Gliem, J. A., & Gliem, R. R. (2003). Calculating, interpreting, and reporting Cronbach’s alpha reliability coefficient for Likert-type scales. In: Midwest Research-to-Practice Conference in Adult, Continuing, and Community Education, p. 82–88.

[39] Koo TK, Li MY (2016). A guideline of selecting and reporting Intraclass correlation coefficients for reliability research. Journal of Chiropractic Medicine.

[40] Scott, D., Ferguson, G. D., & Jelsma, J. (2017). The use of the EQ-5D-Y health related quality of life outcome measure in children in the Western Cape , South Africa : psychometric properties , feasibility and usefulness - a longitudinal , analytical study. *Health and Quality of Life Outcomes*, 1–14. Available from. 10.1186/s12955-017-0590-3. 10.1186/s12955-017-0590-3 PMC5248508 28103872

[41] Burström K, Bartonek A, Broström EW, Sun S, Egmar AC (2014). EQ-5D-Y as a health-related quality of life measure in children and adolescents with functional disability in Sweden: testing feasibility and validity. Acta Paediatrica.

[42] Varni JW, Limbers CA, Neighbors K, Schulz K, JEC L, Heffer RW (2011). The PedsQL™ Infant Scales: feasibility, internal consistency reliability, and validity in healthy and ill infants. Quality of Life Research.

[43] Akoglu H (2018). User’s guide to correlation coefficients. Turkish Journal of Emergency Medicine.

[44] McDougall J, Wright V, Rosenbaum P (2010). The ICF model of functioning and disability: Incorporating quality of life and human development. Developmental Neurorehabilitation.

[45] Mokkink LB, Prinsen CAC, Bouter LM, de Vet HCW, Terwee CB (2016). The COnsensus-based standards for the selection of health measurement INstruments (COSMIN) and how to select an outcome measurement instrument. Brazilian Journal of Physical Therapy.

[46] Myatt HM, Myatt RA (1998). The development of a paediatric quality of life questionnaire to measure post-operative pain following tonsillectomy. International Journal of Pediatric Otorhinolaryngology.

[47] Pillai Ridell R, Fitzgerald M, Slater R, Stevens B, Johnston C, Campbell-Yo M (2016). Using only behaviours to assess infant pain : a painful compromise ?. Pain..

[48] Canaway AG, Frew EJ (2013). Measuring preference-based quality of life in children aged 6–7 years : a comparison of the performance of the CHU-9D and EQ-5D-Y — the WAVES Pilot Study. Quality of Life Research.

[49] Wille N, Badia X, Bonsel G, Burstrom K, Cavrini G, Devlin N (2010). Development of the EQ-5D-Y : a child-friendly version of the EQ-5D. Quality of Life Research.

[50] Eidt-Koch D, Mittendorf T, Greiner W (2009). Cross-sectional validity of the EQ-5D-Y as a generic health outcome instrument in children and adolescents with cystic fibrosis in Germany. BMC Pediatrics.

[51] Bergfors S, Åström M, Burström K, Egmar A-C (2015). Measuring health-related quality of life with the EQ-5D-Y instrument in children and adolescents with asthma. Acta Paediatrica.

[52] Scalone L, Tomasetto C, Matteucci MC, Selleri P, Broccoli S, Pacelli B (2011). Assessing quality of life in children and adolescents : Development and validation of the Italian version of the EQ-5D-Y. Italian Journal of Public Health.

[53] Benjasuwantep B, Chaithirayanon S, Eiamudomkan M (2013). Feeding problems in healthy young children: Prevalence, related factors and feeding practices. Pediatric Reports.

[54] Singendonk M, Goudswaard E, Langendam M, Van Wijk M, Van Etten-Jamaludin F, Benninga M (2019). Prevalence of Gastroesophageal reflux disease symptoms in infants and children: a systematic review. Journal of Pediatric Gastroenterology and Nutrition.

[55] Saigal S, Rosenbaum P, Stoskopf B, Hoult L, Furlong W, Feeny D (2005). Development, reliability and validity of a new measure of overall health for pre-school children. Quality of Life Research.

[56] Holloway JM, Long TM (2019). The interdependence of motor and social skill development: influence on participation. Physical Therapy.

[57] Diamond A (2007). Interrelated and interdependent. Developmental Science.

[58] Mazure A, Szcsepanski T (2013). Pain management in children. Annals of Agricultural and Environmental Medicine.

[59] Liu YH, Stein M (2013). Feeding behaviour of infants and young children and its impact on child psychosocial and emotional development. Encyclopedia Early Childhood Development.

[60] Porter N, Ispa JM (2013). Mothers’ online message board questions about parenting infants and toddlers. Journal of Advanced Nursing.

[61] Van Der Horst K, Sleddens EFC (2017). Parenting styles, feeding styles and foodrelated parenting practices in relation to toddlers’ eating styles: A cluster-analytic approach. PLoS One.

[62] Albers CA, Grieve AJ (2007). Bayley Scales of Infant and toddler development, Third Edition. Journal of Psychoeducational Assessment.

[63] Kim EY, Sung IK (2007). The ages and stages questionnaire: Screening for developmental delay in the setting of a pediatric outpatient clinic. Korean Journal of Pediatrics.

[64] Knox V (2008). Do parents of children with cerebral palsy express different concerns in relation to their child’s type of cerebral palsy, age and level of disability?. Physiotherapy..

